# Genomic BLUP including additive and dominant variation in purebreds and F1 crossbreds, with an application in pigs

**DOI:** 10.1186/s12711-016-0185-1

**Published:** 2016-01-29

**Authors:** Zulma G. Vitezica, Luis Varona, Jean-Michel Elsen, Ignacy Misztal, William Herring, Andrès Legarra

**Affiliations:** GenPhySE (Génétique, Physiologie et Systèmes d’Elevage), Université de Toulouse, INP, ENSAT, 31326 Castanet-Tolosan, France; GenPhySE (Génétique, Physiologie et Systèmes d’Elevage), INRA, 31326 Castanet-Tolosan, France; Departamento de Anatomía, Embriología y Genética, Universidad de Zaragoza, 50013 Saragossa, Spain; Instituto Agroalimentario de Aragón (IA2), 50013 Saragossa, Spain; Animal and Dairy Science, University of Georgia, Athens, GA 30602 USA; PIC North America, 100 Bluegrass Commons Blvd., Suite 2200, Hendersonville, TN 37075 USA

## Abstract

**Background:**

Most developments in quantitative genetics theory focus on the study of intra-breed/line concepts. With the availability of massive genomic information, it becomes necessary to revisit the theory for crossbred populations. We propose methods to construct genomic covariances with additive and non-additive (dominance) inheritance in the case of pure lines and crossbred populations.

**Results:**

We describe substitution effects and dominant deviations across two pure parental populations and the crossbred population. Gene effects are assumed to be independent of the origin of alleles and allelic frequencies can differ between parental populations. Based on these assumptions, the theoretical variance components (additive and dominant) are obtained as a function of marker effects and allelic frequencies. The additive genetic variance in the crossbred population includes the biological additive and dominant effects of a gene and a covariance term. Dominance variance in the crossbred population is proportional to the product of the heterozygosity coefficients of both parental populations. A genomic BLUP (best linear unbiased prediction) equivalent model is presented. We illustrate this approach by using pig data (two pure lines and their cross, including 8265 phenotyped and genotyped sows). For the total number of piglets born, the dominance variance in the crossbred population represented about 13 % of the total genetic variance. Dominance variation is only marginally important for litter size in the crossbred population.

**Conclusions:**

We present a coherent marker-based model that includes purebred and crossbred data and additive and dominant actions. Using this model, it is possible to estimate breeding values, dominant deviations and variance components in a dataset that comprises data on purebred and crossbred individuals. These methods can be exploited to plan assortative mating in pig, maize or other species, in order to generate superior crossbred individuals in terms of performance.

## Background

Crossbreeding schemes are widely used in animal and plant breeding for the purpose of exploiting the heterosis and breed complementarity that often occur in crosses [1]. The main goal of crossbreeding is to improve the performance of crossbred populations. Pure breed/line performance is an imperfect predictor of crossbred performance; there are two reasons that explain this incomplete correlation between purebred and crossbred populations. First, phenotypic measurements on purebred/line individuals are often recorded in only one environment (*e.g.,* management) that differs from the environment in which the crossbred individuals are raised (genotype-by-environment interaction). Second, non-additive genetic effects, such as dominance and/or epistasis, which likely determine heterosis, may result in different breeding values between purebreds and crossbreds.

In the case of dominant inheritance, the theory of pedigree-based genetic evaluation and estimation of genetic parameters for crossbred populations was proposed by Lo et al. [2, 3]. In this model, each individual has two genetic values, one on the purebred scale and one on the crossbred scale. In the absence of inbreeding, it is necessary to estimate nine genetic variance components for an $F_{1}$ cross between breeds/lines (A and B): additive variance for breed A, dominance variance for breed A, additive variance for breed B, dominance variance for breed B, additive variance for the $F_{1}$ population due to the effects of the alleles inherited from breed A, additive variance for the $F_{1}$ population due to the effects of the alleles inherited from breed B, the dominance variance for the $F_{1}$ population, additive covariance between a parent from breed A and an $F_{1}$ offspring, and the additive covariance between a parent from breed B and an $F_{1}$ offspring. Although the relevance of the crossbred model has been shown [4, 5], its use in applied breeding programs is limited, because pedigree relationships between purebred and crossbred individuals are often not known, and large datasets on crosses are needed [6].

Genomic approaches offer tools that allow to perform much deeper analyses, and thus, to understand the effects and the mechanisms of the genes and their interactions that underlie complex traits and to explore new directions for their improvement [7]. In addition, genomic evaluation renews the interest in crossbred individuals because they can be used as training animals [8]. In the case of additive inheritance, a joint genomic evaluation of purebred and crossbred individuals was proposed [9]. Toosi et al. [10] and Zeng et al. [11] extended this approach in order to include dominance. All these studies focused on the selection of purebred individuals for crossbred performance. However, the formal definition of substitution effects and dominant deviations and the estimation of genetic variance components in two breeds/lines and the $F_{1}$ population have not been revisited so far within the genomic framework. This is needed for correct genetic evaluation and for planning selection schemes. The additive variances due to the gametes from the pure lines that compose the $F_{1}$ population are an indicator of how much can be gained by selection. Estimation of dominance variance for the $F_{1}$ individuals can be considered as a predictor of the variability of specific combining ability, i.e. how relevant is assortative mating between purebred lines to maximize the phenotype at a trait of interest in the $F_{1}$ population. As an example, a common procedure in maize breeding is to use “testers” to evaluate the performance of a pure line as a parent in a cross. If the level of dominance variance is high, the use of testers might severely bias selection towards those lines that combine adequately with a particular tester. In practice, the estimated variance components serve as a guide for choosing breeds/lines with good combining abilities (*e.g.,* pigs, corn, etc.) in animal and plant breeding schemes.

The objective of this work was twofold. First, we decomposed variance components for an $F_{1}$ population using a genomic model with additive and non-additive (dominance) inheritance. Second, we applied the approach to estimate variance components using pig data. To our knowledge, there is no published description of the theoretical variance components (additive and dominant) in terms of substitution effect across two pure populations and the crossbred population. The next section describes the theory on which the estimation of genotypic values is based using GBLUP.

## Theory

An $F_{1}$ population involves gametes from the parental populations 1 and 2. If dominance is present, and because allelic frequencies differ in each breed, the within-breed (additive) substitution effects are not equal to the substitution effects across the $F_{1}$ population. Thus, purebred individuals have different breeding values depending on whether they are mated to individuals from the same or another breed/line. This situation is well known [3, 12, 13], and holds even if the genotype effects are constant across breeds or crossbred individuals.

Consider one locus/gene and two non-inbred populations, $P_{1}$ and $P_{2}$ that are each in Hardy–Weinberg equilibrium. An individual from $P_{1}$ is crossed with a random individual from $P_{2}$. Individuals in the $F_{1}$ population have genotypes $B_{1} B_{2}$, $B_{1} b_{2}$, $b_{1} B_{2}$ or $b_{1} b_{2}$ where subscripts 1 and 2 indicate the origin of the allele, i.e. populations 1 or 2, respectively. The genotypic value $G$ of an individual in the crossbred population $F_{1}$ is equal to:

$$G_{{B_{1} B_{2} }} = a,\;G_{{B_{1} b_{2} }} \,{\text{and}}\,G_{{B_{2} b_{1} }} = d \,{\text{and}}\,G_{{b_{1} b_{2} }} = - a ,$$

where $a$ and $d$ are deviations from the midpoint of the two homozygotes, and correspond to the (biological) additive and dominant effects of the gene, respectively. Let us assume that the genotypic values ($a, d$ and $- a$) are the same in the two parental populations and the crossbred population $F_{1}$ (this assumption will be relaxed later) [1], the genetic mean of the $F_{1}$ population is therefore:

$$E\left( G \right) = \left( {pp^{\prime} - qq^{\prime}} \right)a + \left( {pq^{\prime} + qp^{\prime}} \right)d,$$

where $p$ and $q = 1 - p$ are the allelic frequencies of $B_{1}$ and $b_{1}$ in population 1, and $p^{\prime}$ and $q^{\prime}$ are the allelic frequencies of $B_{2}$ and $b_{2}$ in population 2. If the difference in allele frequencies between the two populations is denoted by $y = p - p^{\prime} = q^{\prime} - q$, the genetic mean is, as in Falconer [1], equal to:

$$E(G) = \left( {p - q - y} \right)a + \left[ {2pq + \left( {p - q} \right)y} \right]d.$$

Following the classical parameterization, the genotypic values of individuals in the $F_{1}$ population are the sum of the additive (or breeding) effects of the gametes that originate from populations $P_{1}$ and $P_{2}$ ($u_{1}$ or $u_{2}$) and a dominant deviation $(v)$ which depends on the combination of alleles received [14]:

1 $$G = E\left( G \right) + u_{1} + u_{2} + v,$$

where $u_{1}$ is the additive effect of a gamete from population 1 combined with a gamete from population 2, which differs from the effect of the gamete within the same population. Thus, $u_{1}$ and $u_{2}$ represent the general combining ability (GCA) of alleles $B_{1}$ or $b_{1}$, and $B_{2}$ or $b_{2}$, whereas $v$ is the specific combining ability (SCA) between alleles $B_{1}$ or $b_{1}$, and $B_{2}$ or $b_{2}$. An equivalent expression that is often used in plant breeding is:

$$G = E(G) + GCA_{i} + GCA_{j} + SCA_{ij} ,$$

where the performance of an individual *i* is evaluated in terms of its average performance when it is crossed with another individual *j* [13].

Additive values $u_{1}$ and $u_{2}$ of the gametes include a substitution effect for each gene. Thus, $\alpha_{1}$ is the additive (or breeding) effect of the gametes from population 1 crossed with population 2, and $\alpha_{2}$ the additive (or breeding) effect of the gametes from population 2 crossed with population 1, which are equal to:

$$\alpha_{1} = a + d\left( {q^{\prime} - p^{\prime}} \right)\quad {\text{and}}\quad \alpha_{2} = a + d\left( {q - p} \right).$$

From the expression, $\sigma_{G}^{2} = E\left( {G^{2} } \right) - \left( {E\left( G \right)} \right)^{2}$, the total genetic variance for the $F_{1}$ population is equal to:

$$\sigma_{G}^{2} = \left( {pq + p^{\prime}q^{\prime}} \right)a^{2} - 2\left( {1 - q - q^{\prime}} \right)\left( {pq^{\prime} + p^{\prime}q} \right)ad + \left( {pq + p^{\prime}q^{\prime} - 4pqp^{\prime}q^{\prime}} \right)d^{2} .$$

We can partition the genetic variance $\sigma_{G}^{2}$ into components due to individual additive value (breeding values, $u$), and dominance deviations ($v$). The additive genetic variance for the $F_{1}$ population is:

$$\sigma_{A}^{2} = \frac{1}{2}\sigma_{{A_{1} }}^{2} + \frac{1}{2}\sigma_{{A_{2} }}^{2} ,$$

where $$\sigma_{{A_{1} }}^{2} = 2pq\left( {\alpha_{1} } \right)^{2}$$ and $$\sigma_{{A_{2} }}^{2} = 2p^{\prime}q^{\prime}\left( {\alpha_{2} } \right)^{2}$$.

The part of variance for each population is:

$$\sigma_{{A_{1} }}^{2} = 2pq\left( {\alpha_{1} } \right)^{2} = 2\left[ {pqa^{2} + 2pq\left( {q^{\prime} - p^{\prime}} \right)ad + pq\left( {q^{\prime} - p^{\prime}} \right)^{2} d^{2} } \right],$$

2 $$\sigma_{{A_{1} }}^{2} = 2pq\left[ {a + \left( {q^{\prime} - p^{\prime}} \right)d} \right]^{2} ,$$

$$\sigma_{{A_{2} }}^{2} = 2p^{\prime}q^{\prime}\left( {\alpha_{2} } \right)^{2} = 2\left[ {p^{\prime}q^{\prime}a^{2} + 2p^{\prime}q^{\prime}\left( {q - p} \right)ad + p^{\prime}q^{\prime}\left( {q - p} \right)^{2} d^{2} } \right],$$

3 $$\sigma_{{A_{2} }}^{2} = 2p^{\prime}q^{\prime}\left[ {a + \left( {q - p} \right)d} \right]^{2}.$$

$$\sigma_{{A_{1} }}^{2}$$$$(\sigma_{{A_{2} }}^{2} )$$ is the variance of the GCA of the alleles of individuals from population 1 crossed to individuals from population 2 (alleles of individuals from population 2 crossed with individuals from population 1) or it can also be considered as the additive variance of gametes inherited from population 1 (from population 2) in the $$F_{1}$$ population as in Lo et al. [3].

The variance of the GCA ($\sigma_{A}^{2}$) is an important parameter to understand if selection of purebred individuals can increase crossbred performance [1]. If variance of the GCA explains a large part of the total genetic variance for the $F_{1}$ population, it means that within-population selection will result in a large increase of the crossbred performance, without resorting to specific matings to create crossbreds with large dominance deviations.

The term $ad$ appears in $\sigma_{G}^{2}$ but is completely embedded in $\sigma_{{A_{1} }}^{2}$ and $\sigma_{{A_{2} }}^{2}$. This term differs from 0 if there is covariance between $a$ and $d$, i.e. if $a$ and $d$ are of the same magnitude and direction or if there is overdominance. This covariance between additive and dominant effects of genes implies the presence of inbreeding depression or heterosis. Different models have been proposed to take the dependency between additive and dominant effects into account [15].

Thus, based on Eq. (2) and (3), we can write the additive variance for the $F_{1}$ population as:

$$\sigma_{A}^{2} = pq\left[ {a + \left( {q^{\prime} - p^{\prime}} \right)d} \right]^{2} + p^{\prime}q^{\prime}\left[ {a + \left( {q - p} \right)d} \right]^{2} .$$

Using this last expression of $\sigma_{A}^{2}$ and the expression of the total genetic variance, i.e. $\sigma_{G}^{2} = \sigma_{A}^{2} + \sigma_{D}^{2}$, the variance for the dominance deviation $(v)$ can be obtained as:

$$\sigma_{D}^{2} = \sigma_{G}^{2} - \sigma_{A}^{2} ,$$

$$\sigma_{D}^{2} = \left[ {pq\left( {1 - 2p^{\prime}q^{\prime}} \right) + (p^{\prime}q^{\prime}(1 - 2pq)} \right]d^{2} - \left[ {pq\left( {1 - 2p^{\prime}} \right) + (p^{\prime}q^{\prime}(1 - 2p)} \right]d^{2},$$

where the first and second terms correspond to the total genetic variance and the breeding value (or GCA) variance, respectively. Thus, the dominance genetic variance or the variance of the SCA is equal to:

4 $$\sigma_{D}^{2} = 4pqp^{\prime}q^{\prime}d^{2} ,$$

which leads to the result obtained for a single population if $p = p^{\prime}$ (*e.g.,* [1]).

If $a$ and $d$ effects are considered as random variables with a covariance of 0 between $a$ and $d$, variance components for the $F_{1}$ population can be obtained from these expressions using markers in a GBLUP context as detailed in the next section.

### Equivalent genomic model based on SNPs

A model including (biological) additive and dominant effects of the SNPs can be written in matrix form for a set of individuals as [16]:

$${\mathbf{y}} = \mathbf{1\mu} + {\mathbf{Za}} + {\mathbf{Wd}} + \varvec{e}\text{,}$$

where ${\mathbf{y}}$ is the phenotypic value of individuals, $\mu$ is the population mean and $\varvec{e}$ is the residual. Additive effect ${\mathbf{a}}$ and dominant effect ${\mathbf{d}}$ vectors are included for each of the SNP markers. The matrix ${\mathbf{Z}} = ({\mathbf{z}}_{1} \ldots {\mathbf{z}}_{{\mathbf{m}}} )$ is equal to 1, 0, −1, for SNP genotypes $BB$, $Bb$ and $bb$, respectively. For the dominant component, ${\mathbf{W}} = ({\mathbf{w}}_{1} \ldots {\mathbf{w}}_{{\mathbf{m}}} )$ is equal to 0, 1, 0 for SNP genotypes $BB$, $Bb$ and $bb$, respectively. This model is general and applies to any population structure (purebred or crossed), as far as effects $a$ and $d$ are assumed constant across populations.

From this genotypic model, we can define $u^{*}$ and $v^{*}$ as the genotypic additive and dominant effects, i.e. the parts that are attributed to the additive and dominance “biological” effects [17, 18] of the markers for the whole population (individuals from populations 1 and 2 and the crossbred population $F_{1}$). Note that ‘biological’ is used here to refer to genotypic additive and dominant values of the SNP, to distinguish it from the traditional treatment of quantitative genetics in terms of “statistical” effects (breeding values and dominance deviations). So for a set of individuals ${\mathbf{u}}^{*} = {\mathbf{Za}}$ and ${\mathbf{v}}^{*} = {\mathbf{Wd}}$. Under standard assumptions, the covariances across genotypic additive values are:

$$Cov\left( {{\mathbf{u}}^{ *} } \right) = {\mathbf{ZZ}}\varvec{'}\sigma_{a}^{2} ,$$

where $\sigma_{a}^{2}$ is the SNP variance for additive component. Then, the normalized matrix is:

$$Cov\left( {{\mathbf{u}}^{ *} } \right) = \frac{{{\mathbf{ZZ}}\varvec{'}}}{{\left\{ {tr\left[ {{\mathbf{ZZ}}\varvec{'}} \right]} \right\}/n}}\sigma_{{A^{*} }}^{2} .$$

The division by $\left\{ {tr\left[ {{\mathbf{ZZ}}\varvec{'}} \right]} \right\}/n$ where *n* is the number of individuals scales the matrix to an average of the diagonal elements equal to 1. This covariance matrix is similar to the classical ${\mathbf{G}}$ matrix of genomic BLUP [19], but with a different variance component i.e. $\sigma_{{A^{*} }}^{2}$, the variance component that is associated to the genotypic additive values (this is not a genetic variance *per se* since it cannot be interpreted as the variance of the population). Based on $\sigma_{{A^{*} }}^{2}$, the SNP variance for the additive component can be obtained as $\sigma_{a}^{2} = \frac{{\sigma_{{A^{*} }}^{2} }}{{\left\{ {tr\left[ {\varvec{ZZ'}} \right]} \right\}/n}}$.

Then, the covariance of genotypic values due to dominance is:

$$Cov\left( {{\mathbf{v}}^{ *} } \right) = \frac{{{\mathbf{WW}}\varvec{'}}}{{\left\{ {tr\left[ {{\mathbf{WW}}\varvec{'}} \right]} \right\}/n}}\sigma_{{D^{*} }}^{2} ,$$

where $\sigma_{{D^{*} }}^{2}$ is the variance component associated to genotypic dominant values. The SNP variance for the dominance component can be obtained as:

$$\sigma_{d}^{2} = \frac{{\sigma_{{D^{*} }}^{2} }}{{\left\{ {tr\left[ {{\mathbf{WW}}\varvec{'}} \right]} \right\}/n}}.$$

Therefore, the genotypic model is an equivalent model, which is useful to go from variance components ($\sigma_{{A^{*} }}^{2}$, $\sigma_{{D^{*} }}^{2}$), with no particular interpretations, to marker variances ($\sigma_{a}^{2} , \sigma_{d}^{2}$).

To estimate SNP variance, additive and dominance genetic variances in the F1 population are obtained from Eqs. (2), (3) and (4) extended to multiple loci. The extension to multiple loci assumes linkage equilibrium and uncorrelated marker effects which are standard assumptions [19]. To estimate additive variances, we also assume a covariance of 0 between $a$ and $d$. Thus, the additive genetic variance due to alleles from population 1 in the $F_{1}$ population can be written as:

5 $$\sigma_{{A_{1} }}^{2} = \mathop \sum \nolimits (2p_{i} q_{i} )\sigma_{a}^{2} + \mathop \sum \nolimits (2p_{i} q_{i} \left( {q_{i}^{\prime } - p_{i}^{\prime } } \right)^{2} )\sigma_{d}^{2} ,$$

and the additive genetic variance due to alleles from population 2 in the $F_{1}$ population as:

6 $$\sigma_{{A_{2} }}^{2} = \mathop \sum \nolimits \left( {2p_{i}^{\prime } q_{i}^{\prime } } \right)\sigma_{a}^{2} + \mathop \sum \nolimits \left( {2p_{i}^{\prime } q_{i}^{\prime } \left( {q_{i} - p_{i} } \right)^{2} } \right)\sigma_{d}^{2} .$$

This equation is the variance of GCA among individuals from population 2 crossed with individuals from population 1. It should be recalled that the additive genetic variance for the $F_{1}$ population is equal to:

$$\sigma_{A}^{2} = \frac{1}{2}\sigma_{{A_{1} }}^{2} + \frac{1}{2}\sigma_{{A_{2} }}^{2} .$$

We can also write the dominance genetic variance for the $F_{1}$ population as:

7 $$\sigma_{D}^{2} = \mathop \sum \nolimits (4p_{i} q_{i} p_{i}^{\prime } q_{i}^{\prime } )\sigma_{d}^{2} .$$

For the additive and dominance genetic variances in the parental breeds/lines, expressions are in Vitezica et al. [18]. For instance, for population 1 $(P_{1} )$ with allele frequencies $p$ and $q$, variances are equal to:

$$\sigma_{{A_{{P_{1} }} }}^{2} = \mathop \sum \nolimits (2p_{i} q_{i} )\sigma_{a}^{2} + \mathop \sum \nolimits (2p_{i} q_{i} \left( {q_{i} - p_{i} } \right)^{2} )\sigma_{d}^{2} ,$$

and

$$\sigma_{{D_{{P_{1} }} }}^{2} = \mathop \sum \nolimits \left( {2p_{i} q_{i} } \right)^{2} \sigma_{d}^{2} .$$

Therefore, this approach allows to estimate variance components for the $F_{1}$ population under a genomic model with additive and non-additive (dominance) inheritance. The three variance components in Eqs. (5), (6) and (7) do have an interpretation in terms of variances of breeding values (or GCA) and of dominant deviations (or SCA).

The biological additive and dominant effects of SNPs may not be the same across the different populations, due to genotype by environment or genotype by genotype (i.e. epistasis) interactions.

A simple alternative is to model marker effects as correlated across populations [20], which implies correlated ${\mathbf{u}}^{ *}$ and ${\mathbf{v}}^{ *}$ [21, 22]. This generalizes the methods above.

## Methods

In this section, we illustrate the partition of variance components (additive and dominant) across two pig lines 1 and 2 and the crossbred population. Data for this study were provided by Genus plc (Hendersonville, TN, USA). Animal Care and Use Committee approval was not obtained for this study because the data were obtained from an existing database.

Lines 1 and 2 were two unrelated lines, and population 12 consisted of both reciprocal crosses of animals from lines 1 and 2. Data on litter size (total number of piglets born per litter) were analyzed. The average litter size was equal to 12.68 ± 3.07, 13.15 ± 3.20 and 13.64 ± 3.16 for lines 1 and 2 and population 12, respectively. A total of 34,753 records were available for 8265 sows. Genotypes for all sows were generated using the Illumina PorcineSNP60 BeadChip (Illumina, San Diego, CA). After quality control, i.e. after excluding genotypes with a minor allele frequency lower than 0.05 and a SNP call rate less than 0.90 in the overall population, 40,634 SNPs remained and were used to build genomic relationship matrices. Animals with a call rate less than 0.90 were removed. Thus, the number of sows with genotypes was equal to 3509, 2706 and 2050 in lines 1 and 2 and population 12, respectively.

Phenotypes were collected for the genetic nucleus (pure lines) and commercial herds (crosses). Records were analyzed using a GBLUP (mixed) model. Fixed effects included parity order, farm, year and month of farrowing, and mating type (artificial insemination or natural service).

To estimate the variance components, lines 1 and 2 and population 12 were considered as three different traits with correlations between pure and cross lines [3]. This model is equivalent to a model where marker effects are correlated across populations [20–22] and assumes that additive and dominant effects of a gene ($$a_{1} , a_{2} , a_{12}$$ and $$d_{1} , d_{2} , d_{12} )$$ are not necessarily the same in the three populations. Quantitative trait loci (QTL) that segregate in different breeds are not necessarily identical. In addition, linkage disequilibrium between SNPs and QTL can differ between populations. Even with causal genes, the effects may differ, which was confirmed by experimental results. One example is the bovine *myostatin* gene (*GDF8*), i.e. both the Belgian Blue and South Devon breeds carry the same *GDF8* mutation, but they have different conformation and double-muscling phenotypes [23]. Functional mutations in the *GDF8* gene appear to be breed-specific [24]. Effects can be population-specific and the variation can be interpreted as a dependency of the gene effect on the environmental (GxE) and genetic (i.e. epistasis) backgrounds. Parental pure lines and the $$F_{1}$$ population have only half of their genetic background in common.

In order to estimate the genetic parameters (additive and dominant variances) for the $F_{1}$ population based on SNPs, the multivariate model that includes purebred and crossbred performances was as follows:

$${\mathbf{y}} = {\mathbf{X}}\mu + {\mathbf{u}}^{*} + {\mathbf{v}}^{*} + {\mathbf{p}} + {\mathbf{e}},$$

where $\mu$ is the population mean, ${\mathbf{u}}^{*}$ and ${\mathbf{v}}^{ *}$ are the genotypic additive and dominant effects, ${\mathbf{p}}$ is the permanent environmental effect and ${\mathbf{e}}$ is the residual. The covariance matrix for additive effects is expressed as:

$$Var\left[ {\begin{array}{*{20}c} {{\mathbf{u}}_{1}^{*} } \\ {{\mathbf{u}}_{2}^{*} } \\ {{\mathbf{u}}_{12}^{*} } \\ \end{array} } \right] = {\mathbf{G}}_{\text{o}} \varvec{ } \otimes \;{\mathbf{G}},$$

where ${\mathbf{G}}$ is a normalized genomic additive relationship matrix constructed as ${\mathbf{G}} = \frac{{{\mathbf{ZZ}}\varvec{'}}}{{\left\{ {tr\left[ {{\mathbf{ZZ}}\varvec{'}} \right]} \right\}/n}}$; ${\mathbf{Z}}$ contains values of $\left\{ {1,0, - 1} \right\}$ for each genotype; and ${\mathbf{G}}_{\text{o}}$ is a 3 × 3 covariance matrix as follows:

$${\mathbf{G}}_{\text{o}} = \left[ {\begin{array}{*{20}c} {\sigma_{{A_{1}^{*} }}^{2} } & {\sigma_{{A_{1}^{*} A_{2}^{*} }} } & {\sigma_{{A_{1}^{*} A_{12}^{*} }} } \\ {\sigma_{{A_{1}^{*} A_{2}^{*} }} } & {\sigma_{{A_{2}^{*} }}^{2} } & {\sigma_{{A_{2}^{*} A_{12}^{*} }} } \\ {\sigma_{{A_{1}^{*} A_{12}^{*} }} } & {\sigma_{{A_{2}^{*} A_{12}^{*} }} } & {\sigma_{{A_{12}^{*} }}^{2} } \\ \end{array} } \right],$$

with the variances for the pure lines and the $F_{1}$ population on the diagonal, and the covariances between purebred and crossbred additive effects on the off-diagonals. It should be noted that these variances are not the genetic variances of the populations (lines 1 and 2 and population 12). Based on these variances, it is possible to obtain the SNP additive variance of each pure line $(\sigma_{{a_{1} }}^{2} , \sigma_{{a_{2} }}^{2} )$ and the $F_{1}$ population $(\sigma_{{a_{12} }}^{2} )$*e.g.,* as:

$$\sigma_{{a_{1} }}^{2} = \hat{\sigma }_{{A_{1}^{*} }}^{2} /\left( {\left\{ {tr\left[ {{\mathbf{ZZ^{\prime}}}} \right]} \right\}/n} \right).$$

The covariance matrix for dominant effects is as follows:

$$Var\left[ {\begin{array}{*{20}c} {{\mathbf{v}}_{1}^{*} } \\ {{\mathbf{v}}_{2}^{*} } \\ {{\mathbf{v}}_{12}^{*} } \\ \end{array} } \right] = {\mathbf{D}}_{\text{o}} \otimes \;{\mathbf{D}},$$

where ${\mathbf{D}}$ is a normalized genomic dominant relationship matrix constructed as indicated above with ${\mathbf{D}} = \frac{{{\mathbf{WW}}\varvec{'}}}{{\left\{ {tr\left[ {{\mathbf{WW}}'} \right]} \right\}/n}}$, ${\mathbf{W}}$ contains values of $\left\{ {0,1,0} \right\}$ for each genotype, and ${\mathbf{D}}_{\text{o}}$ is:

$${\mathbf{D}}_{\text{o}} = \left[ {\begin{array}{*{20}c} {\sigma_{{D_{1}^{*} }}^{2} } & {\sigma_{{D_{1}^{*} D_{2}^{*} }} } & {\sigma_{{D_{1}^{*} D_{12}^{*} }} } \\ {\sigma_{{D_{1}^{*} D_{2}^{*} }} } & {\sigma_{{D_{2}^{*} }}^{2} } & {\sigma_{{D_{2}^{*} D_{12}^{*} }} } \\ {\sigma_{{D_{1}^{*} D_{12}^{*} }} } & {\sigma_{{D_{2}^{*} D_{12}^{*} }} } & {\sigma_{{D_{12}^{*} }}^{2} } \\ \end{array} } \right].$$

SNP dominance variances $(\sigma_{{d_{1} }}^{2} , \sigma_{{d_{2} }}^{2} ,\sigma_{{d_{12} }}^{2} )$ are obtained similarly, *e.g.,* as $\sigma_{{d_{1} }}^{2} = \hat{\sigma }_{{D_{1}^{*} }}^{2} /\left( {\left\{ {tr\left[ {{\mathbf{WW^{\prime}}}} \right]} \right\}/n} \right)$. The covariance matrices for permanent environmental and residual effects are as follows: $$ Var\left[ {\begin{array}{*{20}c} {{\mathbf{p}}_{1} } \\ {{\mathbf{p}}_{2} } \\ {{\mathbf{p}}_{12} } \\ \end{array} } \right] = \left[ {\begin{array}{*{20}c} {\sigma_{{p_{1} }}^{2} } & {\bf 0} & {\bf 0} \\ {\bf 0} & {\sigma_{{p_{2} }}^{2} } & {\bf 0} \\ {\bf 0} & {\bf 0} & {\sigma_{{p_{12} }}^{2} } \\ \end{array} } \right]\varvec{ } \otimes \;{\mathbf{I}}_{3} $$, and $$Var\left[ {\begin{array}{*{20}c} {{\mathbf{e}}_{1} } \\ {{\mathbf{e}}_{2} } \\ {{\mathbf{e}}_{12} } \\ \end{array} } \right] = \left[ {\begin{array}{*{20}c} {\sigma_{{e_{1} }}^{2} } & {\bf 0} & {\bf 0} \\ {\bf 0} & {\sigma_{{e_{2} }}^{2} } & {\bf 0} \\ {\bf 0} & {\bf 0} & {\sigma_{{e_{12} }}^{2} } \\ \end{array} } \right]\varvec{ } \otimes \;{\mathbf{I}}_{3}$$, respectively.

Inbreeding was included in the model as a covariate. A molecular metrics of inbreeding, defined as the proportion of genotyped SNPs at which an individual is homozygous [25], was used. It was calculated as the within-individual average homozygosity $(F_{Ho} )$ across all SNPs using the following formula:

$$F_{Ho} = \frac{{N_{AA} + N_{aa} }}{{N_{AA} + N_{Aa} + N_{aa} }},$$

where $N_{AA}$, $N_{Aa}$ and $N_{aa}$ refer to the numbers of SNPs that are classified as *AA*, *Aa*, and *aa*, respectively.

Variance components for the genomic model (GBLUP model) and for a pedigree-based model (PED model, not including dominance) were estimated by EM-REML (expectation maximization restricted maximum likelihood) using the software remlf90 ([26]; available at http://nce.ads.uga.edu/wiki/doku.php), plus an additional iteration of AIREML to obtain the average information matrix. It should be noted that estimated values of $\sigma_{{A^{*} }}^{2}$ and $\sigma_{{D^{*} }}^{2}$ have *per* se no meaningful genetic interpretation.

Additive and dominance variance components at the SNP level ($\sigma_{{a_{1} }}^{2} , \sigma_{{a_{2} }}^{2} ,\sigma_{{a_{12} }}^{2}$ and $\sigma_{{d_{1} }}^{2} ,\sigma_{{d_{2} }}^{2} ,\sigma_{{d_{12} }}^{2}$) were back-solved (dividing by $\left\{ {tr\left[ {{\mathbf{ZZ}}^{'} } \right]} \right\}/n)$ and $\left\{ {tr\left[ {{\mathbf{WW}}\varvec{'}} \right]} \right\}/n$) from variance component estimates ($\sigma_{{A_{1}^{*} }}^{2} ,\sigma_{{A_{2}^{*} }}^{2} ,\sigma_{{A_{12}^{*} }}^{2}$ and $\sigma_{{D_{1}^{*} }}^{2} ,\sigma_{{D_{2}^{*} }}^{2} ,\sigma_{{D_{12}^{*} }}^{2}$, respectively) for the three populations. Genetic variance components for the $F_{1}$ population were obtained from Eqs. (5), (6) and (7). Asymptotic standard errors of variance component estimates were obtained as in [27].

## Results and discussion

### Heritability

To verify whether the correct genetic parameters could be estimated using our approach, the heritability estimates obtained with the traditional pedigree-based model, PED, were compared to those obtained using the genomic GBLUP model. Narrow-sense (additive) heritability coefficients estimated within-line for litter size are in Table 1.

**Table 1:** Narrow-sense heritabilities for litter size in pure pig lines under pedigree-based (PED) and genomic multiple-trait (GBLUP) models

Model	Line 1	Line 2
PED	0.101 ± 0.019	0.102 ± 0.021
GBLUP	0.094 ± 0.014	0.103 ± 0.015

Estimated heritability coefficients across models (PED *vs*. GBLUP) were similar. They were close to 0.10 and consistent with those reported by Nielsen et al. [28] and Guo et al. [29]. Our estimated heritability coefficients for total number of piglets born per litter were also consistent with the average heritability (0.11) reported in the review by Rothschild and Bidanel [30].

### Genetic variances

Additive and dominance variance components that were estimated for pure lines and the $F_{1}$ population are in Table 2. Results show how important it is to estimate the variances for the $F_{1}$ population and point out that within-line variances cannot be directly related to variances for the $F_{1}$ population. Estimates of dominance variance for litter size based on pedigree data have been reported in the literature [6, 31, 32] and were equal to 25 % of the estimated additive genetic variance for litter size. With our genomic model, dominance variance for litter size, was equal to about 15 % of the additive genetic variance and was reasonably consistent with the pedigree-based estimates reported in the literature. Dominance variance for the $F_{1}$ population represents only a small fraction of the total genetic variance i.e. 13 %, which agrees with the results obtained by Misztal et al. [32]. Dominance variance for litter size was found to be slightly greater for the $F_{1}$ population than for the parental lines. Hence, the common belief that low heritability in the narrow sense of the term can hide clearly higher heritability in the broad sense of the term is not supported by the estimated dominance variances.

**Table 2:** Estimates of variances for litter size obtained with the genomic multiple-trait (GBLUP) model

Population	Within line		Crossbred		Permanent environmental variance	Residual variance
	Additive variance	Dominance variance	Additive variance^a^	Dominance variance^b^		
1	0.81 ± 0.13	0.14 ± 0.09	1.47 ± 0.20		0.63 ± 0.15	7.02 ± 0.12
2	0.92 ± 0.14	0.22 ± 0.12	1.44 ± 0.19		0.37 ± 0.18	7.40 ± 0.14
12			1.45 ± 0.19	0.22 ± 0.14	0.94 ± 0.20	6.84 ± 0.12

^a^Additive variance in $F_{1}$ of alleles inherited from population 1 (or 2), or GCA variance, computed using Eqs. (2) and (3)

^b^Dominance genetic variance in the $F_{1}$ population, or SCA variance, calculated from Eq. (4)

The theory presented in this paper and illustrated with these results makes it possible to estimate breeding values and dominance deviations, and to estimate dominance variance for a crossbred population for different traits. It can also be used for more accurate predictions and to assess the relevance of assortative mating in species such as pigs or maize, in order to increase the performance of offspring.

### Genomic correlations

In the GBLUP model, litter size in pure lines and the $F_{1}$ population was analyzed as three traits using a multiple-trait approach (Table 3). The additive correlation of breeding values between pure lines and the $F_{1}$ population refers to the linear association between breeding values of individuals. Selection within the parental lines without including crossbred performance (*e.g.,* in pigs) implicitly assumes that the additive correlation between pure lines and the $F_{1}$ population is equal to 1. As expected, additive correlations of both lines with the $F_{1}$ population are favorable, although less than 1. These values explain the effectiveness of selection on pure lines in breeding programs. Our results show that selection within line 2 is more effective than within line 1 for crossbred performance.

**Table 3:** Additive genotypic correlation (in italics) and dominance genotypic correlation between pure and $F_{1}$ populations

	Line 1	Line 2	$F_{1}$
*Line 1*	1.00	*0.78* *±* *0.21*	*0.60* *±* *0.14*
*Line 2*	0.54 ± 0.38	1.00	*0.83* *±* *0.15*
$F_{1}$	0.47 ± 0.41	0.59 ± 0.36	1.00

Table 3 presents the additive and dominance genotypic correlations for markers ($a$ and $d$) between pure lines and the $F_{1}$ population. The estimated additive genotypic correlation between lines 1 and 2 was equal to 0.78 (Table 3). This indicates that the biological additive effects of SNPs are similar between these lines. Estimating correlations between nominally unrelated lines may seem strange, but genomic relationships allow this estimation. Similar correlations for milk yield were obtained by Karoui et al. [21] between dairy breeds.

For dominance genotypic correlations (Table 3), the values were low regardless of the population, which indicates that dominant effects differ in each population, and that, in practice, assortative mating between two genotypes that would be profitable within, say, line 1 may not be so profitable in the $F_{1}$ population.

Estimates of inbreeding depression, for which the inbreeding coefficient was calculated as the average homozygosity for litter size, were equal to −12.90 ± 2.29 and −10.74 ± 3.03 for lines 1 and 2, respectively. Estimates of inbreeding depression for pure lines expressed as the change in phenotypic mean per 10 % increase in inbreeding were equal to −1.29 and −1.07 piglets born.

## Conclusions

Assuming that SNP effects are independent of the origin of alleles and that allelic frequencies differ between parental populations, we show that the genetic variance for the $F_{1}$ population includes the biological additive and dominant effects of the gene and a covariance term. Genetic variance can be partitioned into additive variance (due to substitution effects of the parental gametes) and dominance deviations. Breeding values of crossbred individuals are generated by substitution effects, where the effects for each parental line depend on the allele frequencies from the other line. In addition, dominance variance is proportional to the product of the heterozygosities of both lines. If the biological additive and dominant effects of markers are considered random with the covariance between them equal to 0, genetic variance components for the $F_{1}$ population can be obtained using an equivalent GBLUP model based on SNPs. The method presented here allows selection for specific combining ability, i.e. selection of a specific pair of parents to produce superior $F_{1}$ individuals, in a GBLUP evaluation framework. The identification of superior $F_{1}$ individuals between inbred/pure lines is an important focus of research in animals and plants [33].
